# 1662. Perspectives of Caregivers of Children with Community-Acquired Pneumonia in the Emergency Department: A Qualitative Study

**DOI:** 10.1093/ofid/ofad500.1495

**Published:** 2023-11-27

**Authors:** Lara Murphy, Nelson Huang, Sujane Kandasamy, Gita Wahi, Jeffrey Pernica

**Affiliations:** University of Toronto, Toronto, Ontario, Canada; University of Toronto, Toronto, Ontario, Canada; McMaster University, Toronto, Ontario, Canada; McMaster University, Toronto, Ontario, Canada; Department of Pediatrics, McMaster University, Hamilton, ON, Canada

## Abstract

**Background:**

Despite the fact that paediatric community-acquired pneumonia (CAP) is usually caused by viruses, antibiotics are often prescribed. The objective of this study was to understand caregiver perspectives and experiences relating to the treatment of paediatric CAP.

**Methods:**

This was a phenomenologically-informed qualitative study involving interviews with caregivers of young children in Hamilton, Ontario. Caregivers were interviewed with open-ended questions relating to germ theory, the nature of pneumonia, and the role of antibiotic treatment. The principles of conventional content analysis were used to guide the coding and synthesis of the transcribed interviews.

**Results:**

Eleven caregivers were interviewed. Most had a good understanding of the difference between bacteria and viruses and many knew that antibiotics were not effective against all types of infections. Many also stated that there was an increased risk of developing resistance with frequent antibiotic use. However, there were misconceptions that probiotics effectively mitigated antibiotic side effects and few were familiar with the potential long-term impacts of antibiotic use in children. There was variability in the perceived severity of paediatric CAP. For children sick enough to require treatment in the emergency department (ED), some participants thought that antibiotic treatment would be important to prescribe to accelerate recovery, as well as to prevent caregivers from feeling helpless. However, participants also thought it was inappropriate for physicians to prescribe antibiotics solely to make the caregiver feel better. Many caregivers also felt strongly that clinical follow-up and discussions on treatment risks and benefits would be desirable and might counteract feelings of helplessness that might result from being sent home without a prescription.

**Conclusion:**

Recognizing that parents may have misperceptions about antibiotic use for CAP (and may seek antibiotics without strong rationale) can inform clinicians’ efforts to better educate and support caregivers in the ED. Care strategies informed by caregiver experiences can improve parent-provider communication, increase trust in the attending clinician, and reduce antibiotic misuse.

**Disclosures:**

**Jeffrey Pernica, MD, MSc, FRCPC, DTMH**, MedImmune: Grant/Research Support|Merck: Grant/Research Support

